# Integrated Nutrition and Culinary Education in Response to Food Insecurity in a Public University

**DOI:** 10.3390/nu13072304

**Published:** 2021-07-04

**Authors:** Susana L Matias, Jazmin Rodriguez-Jordan, Mikelle McCoin

**Affiliations:** Department of Nutritional Sciences and Toxicology, University of California, Berkeley, CA 94720, USA; jazminrjordan@berkeley.edu (J.R.-J.); mikellem@berkeley.edu (M.M.)

**Keywords:** nutrition education, culinary education, food security, young adults, college students, stress

## Abstract

Food insecurity is an emerging issue for college students. A nutrition course with an integrated teaching kitchen was developed to address this issue at a large public university. We aimed to determine changes in food insecurity and stress among students who took the course. The course consisted of weekly lectures followed by teaching kitchen lab sessions to teach basic nutrition and culinary concepts and expose students to hands-on skill development cooking experiences. Using a pre-post design, enrolled students completed an anonymous online survey at the beginning and the end of the semester. Food security was assessed with the USDA Six-Item Food Security Module; stress was measured using the Perceived Stress Scale (PSS). Pre- and post-data were linked for 171 participants. Paired data statistical analysis comparing the post- vs. the pre-test showed an increase in food security and a decrease in very low security rates (from 48% to 70%, and from 23% to 6%, respectively; *p* < 0.0001), and a decrease on the average PSS score, indicating lower stress (from (Mean ± SD) 19.7 ± 5.9 to 18.1 ± 6.0; *p* = 0.0001). A nutrition and culinary course may be an effective response to food insecurity and could potentially improve students’ wellbeing.

## 1. Introduction

Food insecurity, commonly defined as the lack of consistent access to enough food to support an active, healthy life [1], has become an emerging area of concern in college settings [2]. In 2015, the University of California (UC) conducted an assessment of food security across all 10 campuses. The UC wide study revealed that 48% of undergraduate student respondents experienced food insecurity: 25% reported low food security and 23% reported very low food security (also known as food insecurity with hunger) [3]. The UC system-wide estimate was similar to a recently published weighted food insecurity prevalence (41%) based on a sample of 51 2-y and 4-y colleges throughout the US (including the previously mentioned UC sample), but somewhat higher than the one obtained when only 4-y university students were included (36%) [4]. What is consistent among these rates is that they are much higher than the rate of food insecurity for the overall US population. In 2015, when the UC data was collected, 13% of US households reported food insecurity, including 5% reporting very low food security [5].

The higher food insecurity rate in the college population is concerning because of the negative outcomes associated with food insecurity observed in the general population, such as decreased intake of fruits and vegetables [6] and micronutrients [7], and chronic diseases, including diabetes [8,9,10], hypertension [10], hyperlipidemia [9], and possibly nonalcoholic fatty liver disease [11]. Food insecurity has also been related to overall poor mental health [12] and depression [13] among US women. 

Correlates of food insecurity in college students (vs. food secure students) include more food access barriers [14], a less healthy diet [14,15,16], and lower cooking and food agency [15]. Furthermore, as observed among children [17], food insecurity was also associated with difficulty concentrating and lower academic performance in college students [2,18]. Longitudinal data from seven colleges and universities in Georgia (*n* = 2377) revealed that students’ psychological health may be a mechanism through which food insecurity affects academic performance (i.e., lower GPA) in college students [19]. 

Responses to food insecurity among postsecondary institutions would likely need to include interventions targeted at the intrapersonal, interpersonal, and institutional levels in order to effectively address this issue. As part of an action plan to address food insecurity in a large public university campus, a nutrition course with an integrated teaching kitchen was created and offered to increase nutrition knowledge and provide opportunities for students to develop nutrition and culinary skills. Previously, we reported increased consumption of fruits and vegetables, increased frequency of cooking, and reduced frequency of skipping meals among students after taking the nutrition course [20]. In this analysis, we aimed to determine whether food insecurity rates changed after students took the nutrition course with an integrated teaching kitchen lab. Secondarily, we aimed to determine whether students’ stress levels also changed after participation in the class.

## 2. Materials and Methods

### 2.1. Study Design

We used a pre-post intervention study design to evaluate the intervention, i.e., a 2-unit elective undergraduate nutrition course with an integrated teaching kitchen lab. The intervention consisted of a 14-week (semester-long) course that was developed to address food insecurity among students. The rationale for a nutrition and cooking course as part of the campus efforts to reduce food insecurity is based on the less healthy diet [14,15,16] lower cooking self-efficacy [21] and food agency [15], and less cooking or food preparation [15,21] reported by food insecure college students (versus food secure counterparts).

### 2.2. Intervention

The nutrition course was developed on principles from the Social Cognitive Theory [22] to improve students’ nutrition-related behaviors by addressing knowledge, attitudes, skills, and barriers related to food selection, procurement, and preparation with a focus on intersections with food security. Hands-on education was provided in our department’s teaching kitchen, where students learned through experiential learning and observation, addressed social norms, and engaged in skill building activities. The course consisted of weekly 50 min lectures, led by one of the investigators (JRJ), 2 h faculty and/or graduate student-led cooking classes in a teaching kitchen with nine stations. During the lectures, students were taught about basic nutrition and cooking concepts, meal planning and food budgeting, nutrients and their food sources, dietary guidelines, calculation of energy and nutrient needs, food labels, and mindful eating. A discussion of food insecurity in college, with representation from various local resources (e.g., Supplemental Nutrition Assistance Program (SNAP)—County Educator) was also included. Cooking concepts taught included food storage and safety, and cooking methods utilizing different kitchen equipment (e.g., stovetop, oven, and microwave). During the cooking lab, students worked in pairs at each station to promote problem solving and role modeling in cooking and trying new techniques; each pair of students at each station was assigned at least 2 recipes during the lab depending on the ease and total cooking time. To foster connection and community, students rotated lab partners weekly. Recipes included affordable and nutritious ingredients, were easy to follow, and required less cooking time to promote cooking self-efficacy. Students learned how to cook vegetables, whole grains, legumes, and animal-based proteins, although plant-based meals were emphasized. As part of their coursework, students completed several projects, such an activity involving shopping for groceries to understand the link between nutritional quality and cost of foods, and a week-long meal plan (and its nutrient analysis) on a $50 budget. The purpose of this project was to create a personal meal plan while implementing the acquired knowledge of purchasing and preparing nutrient-dense foods at a cost comparable to the Thrifty Food plan [23], which is used as basis for the SNAP allowance. Further details about the nutrition course have been published [20]. 

### 2.3. Participants and Recruitment

The study was conducted at a large public university in California. In 2017, there were 30,574 undergraduate students, of which 52% were female, 21% were transfer students, 39% identified as Asian and 25% as White. Enrollment for the class was open for undergraduate students at any academic standing (e.g., freshman, sophomore, etc.); however, interested students who were considered at risk of food insecurity were given priority enrollment. This was determined using a 4-question screener that students completed before they could enroll in the class. This analysis is based on data collected over five semesters, between fall 2017 and fall 2019. An explanation of the research, invitation to participate, and revision of the consent form were part of the first teaching kitchen lab session. A consent form was signed before data collection took place. An Institutional Review Board approved the study protocol. 

### 2.4. Data Collection

Participants completed the pre- and post-test online survey at the beginning and at the end of the semester. The anonymous survey was developed using an online platform (Qualtrics, Provo, UT, USA). Students received a link to access the survey and complete it within that platform. The survey included several sociodemographic questions, including age, self-identified gender, and race/ethnicity; students were also asked about previous nutrition coursework, living arrangements (e.g., off campus), and academic standing (e.g., freshman). 

To measure food insecurity, we used the US Household Food Security Survey Module: Six-Item Short Form [24]. This short module has been shown to identify food insecure households and households with very low food security with reasonably high specificity and sensitivity, and minimal bias compared with the 18-item US Household Food Security Survey Module [25]. For this study, we used a timeframe recall of the past 30 days. Food security scores range between 0 and 6, which were categorized as *High or marginal food security* (scores 0–1), *Low food security* (scores 2–4), or *Very low food security* (scores 5–6). The reliability (i.e., internal consistency) of this scale in the study sample was assessed using the Cronbach’s alpha coefficient, which was 0.86 and deemed satisfactory [26]. During the study, changes in the coding of the food security questions implemented in the online platform resulted in some unusable data (*n* = 27). However, using incomplete scoring and an algorithm, the investigators were able to deduce the food security categorical level for some of these cases (*n* = 17).

The online survey also included the Perceived Stress Scale [PSS] [27], a 10-item self-report scale that assesses the degree to which one perceives an event or situation as threatening or demanding and beyond one’s coping resources. The response items are rated on a 5-point Likert scale ranging from 0 (never) to 4 (very often) based on a 30-day recall timeframe. Scores range from 0 to 40, with higher scores indicating greater levels of perceived stress. The PSS has been used in undergraduate college samples [28,29] showing good reliability [28]. Its reliability in the study sample was adequate (i.e., Cronbach’s alpha coefficient = 0.84). 

Additionally, we included a previously validated questionnaire [30] to measure attitudes and self-efficacy for increasing consumption of fruits and vegetables, and for cooking, as well as related behaviors. Details on these assessments and findings focused on dietary and food preparation variables have been published elsewhere [20]. 

### 2.5. Data Analysis

Descriptive statistics (i.e., frequencies and percentages) were used to characterize the sample. Histograms and the Shapiro–Wilk normality test were used to determine whether continuous data were normally distributed. For categorical data (i.e., food security categories), the Bowker’s symmetry test for paired samples [31] was used, which is an extension of the McNemar’s test for paired samples that allows more than two categories. For normally distributed continuous data (i.e., PSS scores), means and standard deviations were calculated for each time point (i.e., pre and post), a paired t-test (Δ = post-pre) was conducted for hypothesis testing, and the corresponding Cohen’s d effect size ((Mean_post_ − Mean_pre_)/SD_pool_) was computed [32]. The following post hoc exploratory analyses were also conducted: (1) Spearman correlations (*r*_S_) between the four previously reported behavior changes [20] of increased intake of fruits, increased intake of vegetables, increased frequency of cooking, and reduced frequency of skipping meals in the post-test (vs. the pre-test), and changes in food security scores, (2) Spearman correlations between changes in food security scores and changes in PSS scores, and (3) Spearman correlations between the four previously reported behavior changes and changes in PSS scores. All hypotheses testing were two-sided and the significance level was set to 5%. All statistical analyses were conducted using SAS version 9.4 (SAS Institute Inc., Cary, NC, USA).

## 3. Results

Out of 216 students enrolled in the course, 99% of them (*n* = 214) enrolled in the study during the five semesters covered in this study. Four hundred and four pre- and post-online surveys were completed during the study period, but 21 of these were excluded due to missing study ID (which prevented linkage of pre- and post-test surveys). Of the 383 surveys remaining, pre- and post-test surveys were linked for 171 (paired) participants. Paired participants were not significantly different from those who remained unpaired (*n* = 41) in age (*p* = 0.74), gender (*p* = 0.25), race/ethnicity (*p* = 0.25), academic year (*p* = 0.44), living arrangements (*p* = 0.74), nutrition coursework (*p* = 0.24), and food security level (*p* = 0.80), but their baseline average PSS score was lower than that of those not paired (*p =* 0.02). Table 1 shows sample characteristics based on the baseline (pre-test) survey data. Most participants were women, identified as non-Hispanic Asians, and lived off-campus. Fifty-two percent reported food insecurity, and about half of them had no previous nutrition coursework.

**Figure 1:**
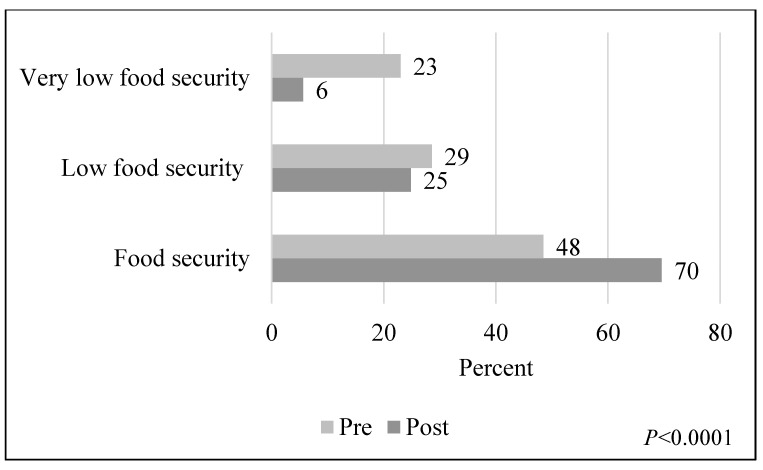
Distribution of food security categories in the pre- and post-test (*n* = 161).

Out of the 171 paired participants, 161 had pre- and post-test food security data and 163 had pre- and post-test PSS scores, corresponding to 75% and 76% of those enrolled in the study, respectively. Figure 1 shows the percent of participants in each food security category in the pre- and post-test. Out of the 161 paired participants with complete food security data, 78 reported food security, 46 reported low food security, and 37 reported very low food security in the pre-test. In the post-test, the number of participants who reported food security increased to 112, while the number of participants who reported low food security and very low food security decreased to 40 and 9, respectively. Based on the Bowker’s symmetry test, changes between the pre- and post-test distributions were statistically significant (*p* < 0.0001).

Among those with complete PSS data, the mean ± SD PSS score was 19.7 ± 5.9 in the pre-test and 18.1 ± 6.0 in the post-test. The mean Δ (post-pre) score was −1.6, with a 95% confidence interval (−2.3, −0.8) that did not include zero (*p* = 0.0001), and the Cohen’s effect size for the perceived stress outcome was −0.27.

**Figure 2:**
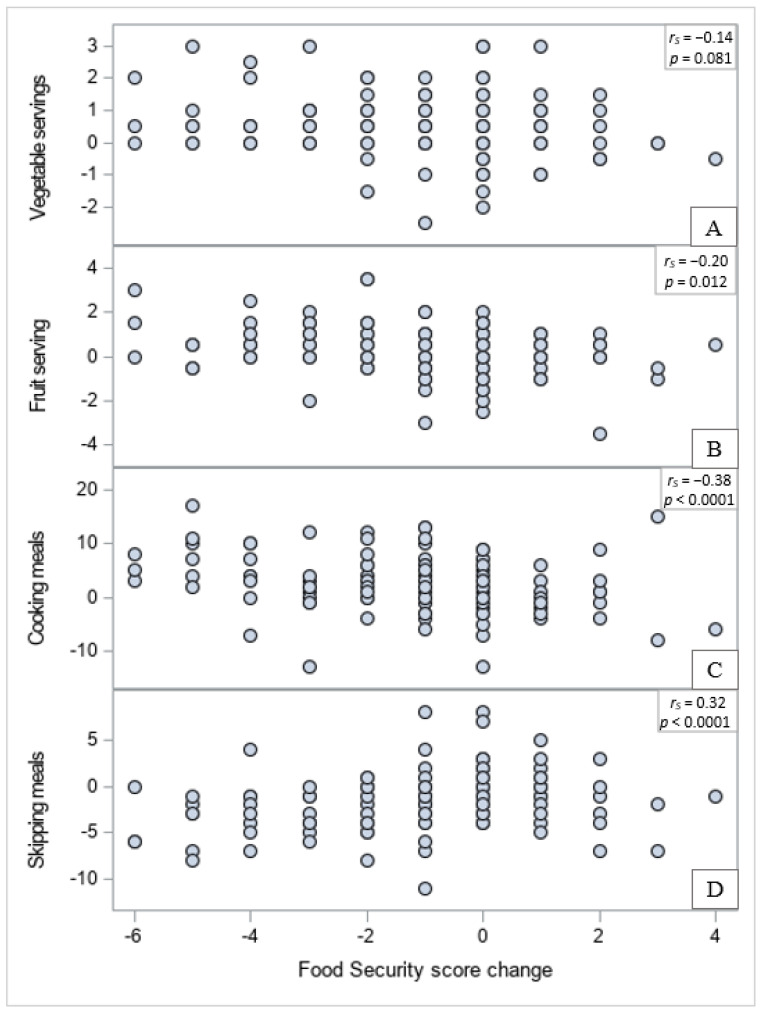
Scatter plots and Spearman correlations (*r*_S_ and *p*-values) between previously reported changes in vegetable intake (**A**), fruit intake (**B**), cooking frequency (**C**), skipping meals frequency (**D**) and changes in food security scores (*n* = 149).

**Figure 3:**
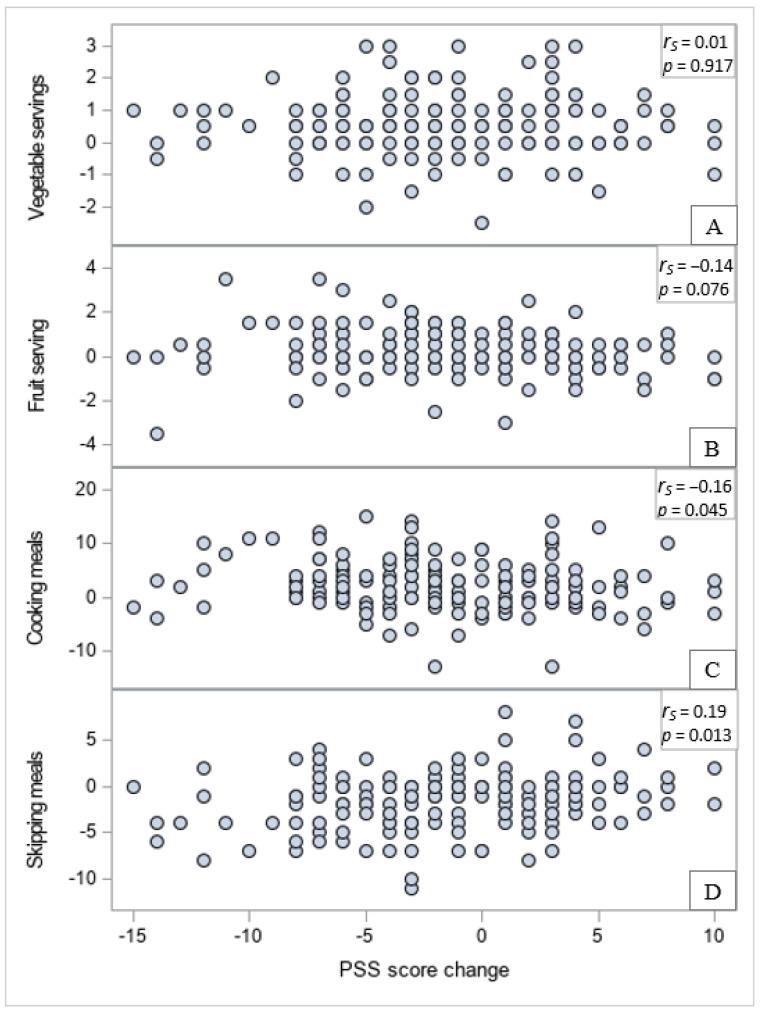
Scatter plots and Spearman correlations (*r*_S_ and *p*-values) between previously reported changes in vegetable intake (**A**), fruit intake (**B**), cooking frequency (**C**), skipping meals frequency (**D**) and changes in the Perceived Stress Scale (PSS) scores (*n* = 163).

Results from post hoc analysis are shown in Figure 2 and Figure 3. Increments in the number of vegetable servings per day were marginally associated with reductions in food security scores (indicating higher food security; Figure 2A; *r*_S_ = −0.14, *p* = 0.08). Increments in the number of fruit servings per day were associated with significant decreases in food security scores (increased food security; Figure 2B; *r*_S_ = −0.20, *p* = 0.01). Stronger associations were observed between changes in food security scores and changes in food preparation behaviors. Reduced food security scores (increased food security) were associated with increased frequency of cooking (Figure 2C; *r*_S_ = −0.38, *p* < 0.0001), and reduced frequency of skipping meals (Figure 2D; *r*_S_ = 0.32, *p* < 0.0001). Changes in food security scores were not related to changes in PSS scores, based on a Spearman correlation coefficient *r*_S_ = −0.00 (*p* = 0.95). Reductions in PSS scores were not significantly associated with changes in vegetable intake (Figure 3A; *r*_S_ = 0.01, *p* = 0.92), but they were negatively associated with changes in fruit intake, although only marginally (i.e., reduction in stress tended to be associated with increased consumption of fruits; Figure 3B; *r*_S_ = −0.14, *p* = 0.08), and with frequency of cooking (i.e., reduction in stress was associated with more cooking; Figure 3C; *r*_S_ = −0.16, *p* = 0.045), and positively associated with frequency of skipping meals (i.e., reduction in stress was associated with less meals skipped; Figure 3D; *r*_S_ = 0.19, *p* = 0.013).

## 4. Discussion

Overall, this study found that participation in a semester-long undergraduate nutrition course with an integrated teaching kitchen lab might contribute to reducing food insecurity and potentially stress levels among undergraduate students, in the context of a large public university. To our knowledge, this is the first report of such potential impact from a nutrition and culinary intervention approach among young adults in a college setting. This is particularly relevant given the high burden of food insecurity [4] and stress [33] in the college student population.

In a qualitative study conducted in another UC campus to understand students’ food insecurity, emergent themes identified students’ desire to improve their food literacy. Food literacy themes that came up in that study included knowledge and skills, enjoyment and social cohesion, and a desire for practical food literacy “life skills” training, including those around food preparation and budgeting [34]. Similarly, in a quantitative study across the UC system, students expressed wanting assistance with learning to cook affordable, healthy meals and to budget with limited resources [3]. It is important to note that the nutrition and culinary course evaluated here addressed those topics. 

In recent studies, food insecurity was associated with lower cooking self-efficacy and agency and, consistently, with less food preparation among college students [15,21]. On the other hand, people with high or moderate levels of cooking, food preparation, and financial skills are less likely to experience food insecurity than people with lower skill levels [35]. Therefore, another potential mechanism by which this nutrition and culinary education may have alleviated food insecurity might be through previously reported increased cooking self-efficacy and more frequent meal preparation [20]. This is supported by the observed significant association between cooking more often and skipping meals less frequently with improved food security. 

In addition to lack of cooking skills, food insecurity has also been linked to unhealthy dietary patterns in college students. At a large, public Midwestern university, low food security was associated with lower intake of fruits and very low food security was associated with higher intakes of added sugar among students [15]. However, as previously reported, students who participated in this nutrition course increased their intake of fruits and vegetables [20]. Findings from the current analysis indicate that those dietary changes were linked to improvements in food security. Accordingly, a nutrition and culinary course, like the one evaluated here, may not only alleviate the students’ food insecurity burden but it may also affect its correlates, potentially improving students’ wellbeing in a more comprehensive manner.

Even more interestingly, after taking the nutrition and culinary course, students’ stress levels were modestly reduced as measured by a validated and widely used scale. A qualitative study conducted with college students from the University of California explored how food insecurity affected psychosocial health. Emergent themes from the interviews included the stress of food insecurity interfering with daily life, resentment of students in more stable food and financial situations, sadness from reflecting on food insecurity, feeling hopeless or undeserving of help, and frustration directed at the academic institution for not providing enough support [36]. Quantitative research in Georgia [19] and California [18] reported a significant association between food security and psychosocial or mental health among college students. However, although food insecurity was related to students’ stress levels at baseline (data not shown), there was no association between change (improvement) in food security and change (reduction) in stress levels. 

Additional analysis into other intervention outcomes indicated that stress reduction correlated with increased fruit intake, although not with consuming more vegetables. A cross-sectional survey with undergraduates in seven universities in England, Wales, and Northern Ireland (*N* = 3706) found that consuming ‘healthy’ foods (e.g., fresh fruits, salads, cooked vegetables) was significantly negatively associated with perceived stress and depressive symptoms scores [37]. In the current study, we also found that reduced stress was significantly associated with behavioral changes in meal preparation. Specifically, students who reported reduced stress levels were cooking more frequently and skipping meals less often. A systematic review of cooking interventions suggested that such interventions may positively influence psychosocial outcomes [38]. Furthermore, in focus groups conducted in another UC campus to explore students’ food insecurity experiences, students mentioned enjoying cooking as a way to relax, relieve stress, and be creative [34]. Thus, findings from this study suggest that a nutrition course with an integrated teaching kitchen, originally developed as a response to food insecurity, may not only be an effective response to this issue in college settings but may result in further positive impacts that could improve students’ emotional wellbeing.

Caution in the interpretation of the findings is advised, as this study had some limitations. The main study limitation is the lack of a control or comparison group. This reduces the ability to attribute the changes reported to the nutrition and cooking course, because it cannot be ruled out that such changes were due to other exposures besides the intervention. In addition, selection bias may have been introduced, as students who chose to enroll in the nutrition class may have been particularly motivated to learn and make behavioral changes that influenced the observed outcomes. Further limitations are the use of a non-random sample, which further limits generalizability of study findings, and the assessment of the outcomes via self-reporting, which may introduce reporting bias. Moreover, some participant losses occurred due to inability to match pre- and post-surveys due to missing study ID (*n* = 41). However, students included were not significantly different from those we could not match in sociodemographic characteristics. This study also has important strengths, such as the inclusion of a socioeconomically and racially diverse sample from a large public university, and the use of a data collection tool (an anonymous online survey) that reduces interviewer effects and social desirability bias [39]. 

Nevertheless, since food insecurity is an increasing problem in college settings and with emergencies (such as the COVID-19 pandemic, wildfires, etc.) resulting in increased food prices, more rigorous evaluations of interventions that combine nutrition and culinary education to address food insecurity are warranted. 

## Figures and Tables

**Table 1:** Sample characteristics (*n* = 171).

Characteristic	*n* (%)
Age	
18–20	89 (52.0%)
21–23	74 (43.3%)
24+	8 (4.7%)
Gender	
Female	107 (62.6%)
Male	62 (36.3%)
Other	2 (1.2%)
Race/ethnicity	
Non-Hispanic White	19 (11.1%)
Non-Hispanic Asian	90 (52.6%)
Hispanic	42 (24.6%)
Non-Hispanic Other	20 (11.7%)
Academic standing	
Freshman	15 (8.8%)
Sophomore	29 (17.0%)
Junior	49 (28.7%)
Senior	74 (43.3%)
Other	4 (2.3%)
Food security status	
Food security	80 (47.9%)
Low food security	48 (28.7%)
Very low food security	39 (23.4%)
Off campus housing	138 (80.7%)
Previous nutrition coursework	84 (49.1%)

## Data Availability

The data presented in this study are available on request from the corresponding author. The data are not publicly available due to ethical approval restrictions.
